# First record of the thread-legged assassin bug *Myiophanes greeni* Distant, 1903 (Heteroptera: Reduviidae: Emesinae) from India

**DOI:** 10.3897/BDJ.4.e7949

**Published:** 2016-03-10

**Authors:** Siddharth Kulkarni, Hemant Ghate

**Affiliations:** ‡ Hemi Terrace Bldg, Near Ellora Palace, Balajinagar, Pune, India; § Modern College of Arts, Science and Commerce, Shivajinagar, Pune, India

**Keywords:** Reduviidae, thread-legged bug, new record, India.

## Abstract

**Background:**

While surveying bugs and spiders in the caves of Satara District, Maharashtra, one of us (SK) collected a thread-legged bug associated with a spider web.

**New information:**

A Sri Lankan Emesinae bug, *Myiophanes greeni* Distant (Heteroptera: Reduviidae: Emesinae) is reported for the first time from India. The species is redescribed with several illustrations including male genitalia.

## Introduction

While surveying true bugs (Heteroptera) and spiders in the caves of Satara District, Maharashtra, India one of us (SK) collected a thread-legged assassin bug (Reduviidae: Emesinae) associated with a spider web. The specimen was identified as *Myiophanes greeni* Distant, 1903. The species was described by Distant (1904) on the basis of material collected at ‘Puttalam, Ceylon (Green)’. This species has so far been known only from type locality. In fact, this species has not been reported again since original description in 1903. Wygodzinsky (1966) placed it in the subgenus *Myiophanes*–therefore the current valid combination of the name is Myiophanes (Myiophanes) greeni Distant, 1903.

The Fauna of British India, Rhynchota volume II was published in two parts; Part 1 (x + 242 pp) was published in 1903 and Part 2 was published in 1904 with a re-issued title-page for the whole II volume, dated 1904. Part 1 contained the whole of the Emesinae, so the date of publication of *Myiophanes greeni* Distant is 1903.

The species is briefly redescribed in the present paper, with digital illustrations of various morphological characters, including pygophore and aedeagus as well as parameres. Because it has not been reported from any part of India before (Ambrose 2006), this represents the first record of *M. greeni* from India and also the northernmost extension of the range of the species. The only species of the genus *Myiophanes* Reuter, 1881 listed by Ambrose (2006) is *M. kempi* China, 1924, from Assam, also a cavernicolous species.

Distant (1904) gave a good account of general coloration along with a habitus drawing, and Wygodzinsky (1966) gave a detailed review of the genus – both of these publications were helpful in identification of this bug. In addition, an image of the type of the specimen, preserved in Natural History Museum, London, was kindly provided by Mick Webb.

## Materials and methods

A live emesine bug was collected from a cave located at Chalkewadi road near Sajjangad fort Satara, Maharashtra, India. It was studied under a Leica microscope (stereozoom MZ6) and photographed with an attached Canon Powershot S50 camera. Multiple images were stacked using the Combine ZM software and the stacked images were processed with Adobe Photoshop CS5. Measurements were done with Erma slide / stage micrometer and an accurate scale. Pygophore was dissected after treating the last three abdominal segments with hot 10% KOH, phallic complex was dissected, parameres and aedeagus were separated and mounted in glycerine for photography under microscope. Genitalia was mounted in Polyvinyl Lacto-Phenol (PVLP) with lignin pink dye, and photographed using Axiocam ER65S attached to a Zeiss microscope. The specimen is preserved at Modern College, Pune, India.

## Taxon treatments

### Myiophanes (Myiophanes) greeni

Distant, 1903

#### Materials

- **Type status:** Other material. **Occurrence:** catalogNumber: Insects; recordedBy: Siddharth Kulkarni; individualCount: 1; sex: male; lifeStage: adult; **Taxon:** scientificName: Myiophanes greeni; kingdom: Animalia; phylum: Arthropoda; class: Insecta; family: Reduviidae; genus: Myiophanes; subgenus: Myiophanes; taxonRank: species; scientificNameAuthorship: Distant; **Location:** country: India; stateProvince: Maharashtra; municipality: Satara; locality: Cave near Sajjangad road; decimalLatitude: 17.618; decimalLongitude: 73.881; **Identification:** identifiedBy: Hemant Ghate; dateIdentified: 2015; **Event:** samplingProtocol: visual searching; eventDate: 10/18/2015; **Record Level:** language: en; institutionID: Zoology Department, Modern College, Shivajinagar, Pune; collectionCode: Insects; basisOfRecord: PreservedSpecimen

#### Description

**General coloration**

**Figure 1.**
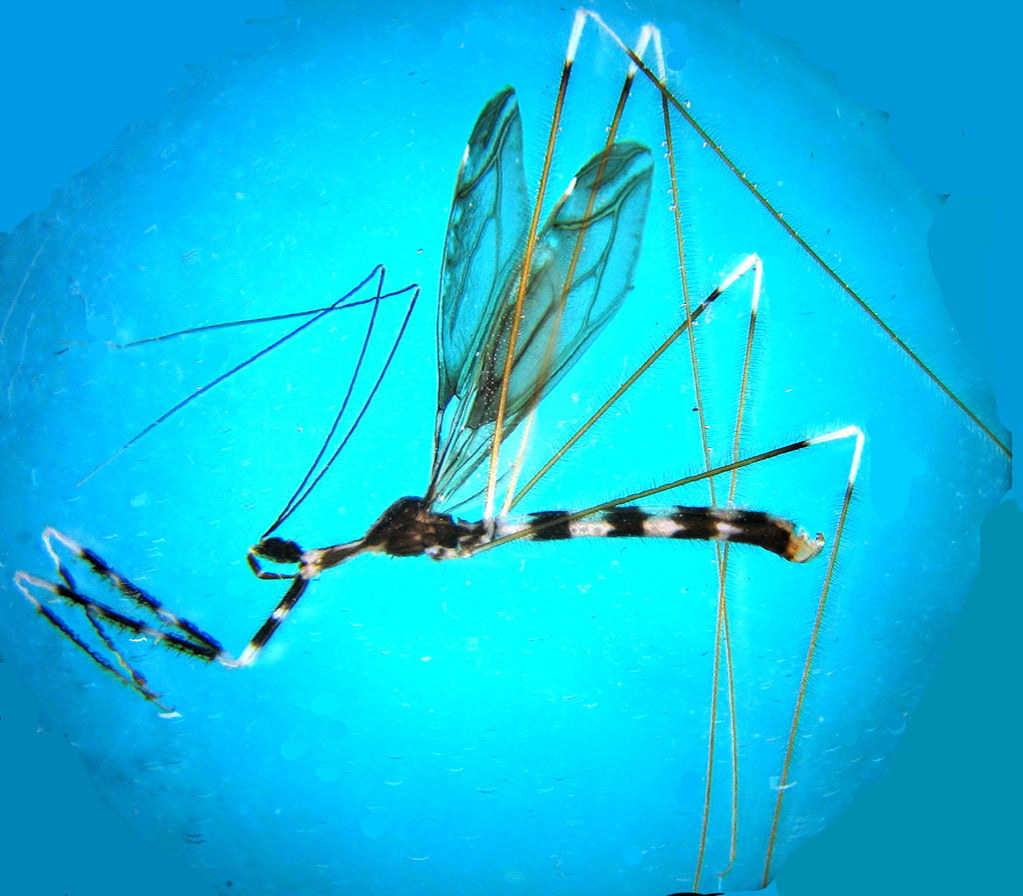
*Myiophanes greeni*, fresh male, lateral view.

With contrasting pattern of dark to pale brown patches and with creamy white areas. Femoro-tibial junction of mid and hind legs, base and distal part of meso- and meta-coxae and a spot on pterostigma (= distal, thickened portion of Sc+R vein) also creamy white. Most of anterior part of anterior lobe of pronotum creamy white, the white coloration of anterior lobe of pronotum extends backward on posterior lobe as a fine pointed stripe on either side of mid-line. Entire pro-sternum creamy white; mesosternum dark brown on disc, on either side of which is a fuscous band bordered with dark brown line beyond which there is another pale band followed by a brown band; metasternum mostly dark brown (Fig. 1). Entire body and legs pilose.

**Morphology**

Head: Dark brown; oblong oval; eyes black, large, protuberant, occupying large part of lateral area; anterior and posterior ocular part almost subequal in length; dorsal surface slightly convex with a deep interocular groove which is paler in colour, than adjacent areas; ventral surface flatter than dorsal side, mostly brown except for pale stripe of either side of mid‐line along eye; neck region also with a pale white patch ventrally close to prosternum (Fig. 2​). Antennal tubercles projecting anteriorly in front of eyes. Clypeus prominent, projecting in front, medially dark brown, laterally pale creamy. Mandibular plates laterally dark brown, with a thin stripe of pale cream adjacent to clypeus. Labium with three visible segments (first visible segment representing morphological segment II); segment II thickened, pale at base and apex, dark brown dorsally and pale brown ventrally; segment III less thick, also dark brown dorsally and pale brown ventrally, apex creamy white; segment IV somewhat curved, longer than segment III, tip reaching between fore-coxae. Entire head covered with numerous dark brown setae.

Antenna brown, segment IV creamy white; with segment I longest, segment II slightly shorter than I,segment III shortest; segment IV long but shorter than I and II; setose, some setae dark brown, otherstranslucent. Prothorax: Pronotum gradually narrowed and then considerably broadened in dorsal view, this constriction marks boundary between anterior (funnel-shaped) and posterior (sub-triangular) lobes. Posterior lobe sloping, its dorsal outline enclosing an angle of about 140 degrees with that of anterior lobein lateral view; dorsal outline of anterior lobe slightly convex, almost flat on middle part of disc, posteriorlobe also more or less flat on disc, but slightly convex at base in lateral view (Fig. 3b​). Humeral angles notprominent. Maximum width of posterior lobe is at base and is slightly more than two times the width atanterior margin. Anterior margin slightly concave behind head. Anterior angles sub-prominent, bluntlytriangular and dark brown. Pronotum extends somewhat downwards laterally to conceal fore coxae whichopen forward but are seen through due to translucent coloration.

Pronotum with most part of anterior lobe creamy white dorsally with two broad lateral stripes and amedian thin stripe in posterior half of anterior lobe brownish; a white stripe on anterior lobe extendsbackward to posterior lobe, one on either side of midline, slightly obliquely and gradually narrowing alonglength ending in a fine tip, just where posterior lobe flattens dorsally. Similarly, a median brown band(with a central thin white line) starting from anterior third of anterior lobe and gradually broadenedanteriorly, meets brown part of posterior lobe. Entire dorsal surface as well as prosternum covered withsparse but very long brown setae. Scutellum triangular, dark brown (Fig. 3​).

**Figure 4.**
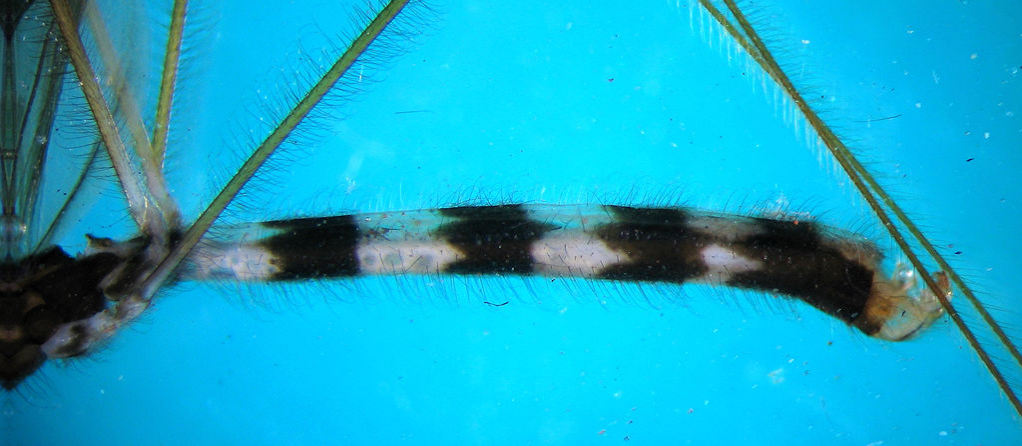
*Myiophanes greeni*, abdomen, lateral view.

Hemelytra and wings as described for the genus by Wygodzinsky (1966). Venations as in (Fig. 1). Abdomen slightly narrow at base, then broadened, rest of sides of abdomen parallel but again narrowed near apex,with an alternating pattern of brown and pale white annuli (Fig. 4). Abdominal segments darker ventrallythan dorsally. Boundaries of tergites not clearly marked from dorsal side, first two brownish patches verypale, third, fourth, fifth darker. Connexivum very narrow, raised upward.

Pygophore dorsally colourless (Fig. 5a​). Tips of parameres and ventromedian, upwardly directed, posteriorspiniform process of pygophore dark brown (Fig. 5b).

Fore legs long, coxae eight times longer than broad, with two broad dark brown to black annuli, one at baseand one beyond middle. Trochanter creamy white. Fore femur almost twelve times as long as broad,creamy white, with three dark brown annuli (one at base, second in middle and third beyond middle) (Fig. 2a​).

**Figure 6.**
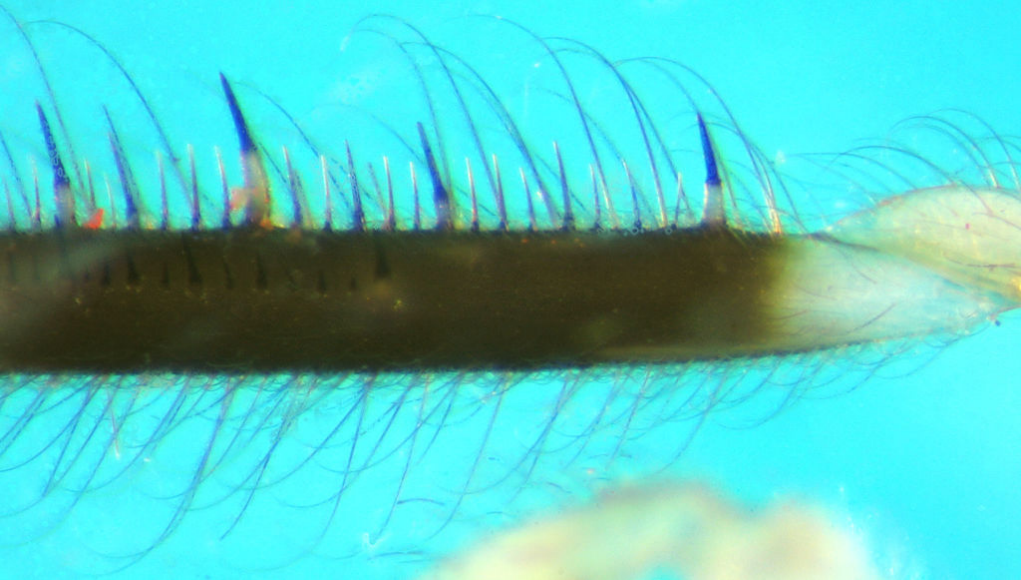
*Myiophanes greeni*,internal (anterior) view of fore femur.

Femur with two rows of spines underneath, one external (posteroventral), one internal (anteroventral);with several relatively long spines with broad white basal half and dark black spiny distal half and manysimilar but smaller and thinner spines; internal row of spines starts slightly distally from base (Fig. 6).Tibia long but slightly shorter than femora, creamy white at base and brownish to fuscous at most of itslength, distal tip slightly swollen, underside with many spiniform setae with short broad base and long blackspiny projections. Tarsus pale creamy, its three segments more or less sub‐equal in length. Claws partly pale brown (Fig. 7).

**Figure 9.**
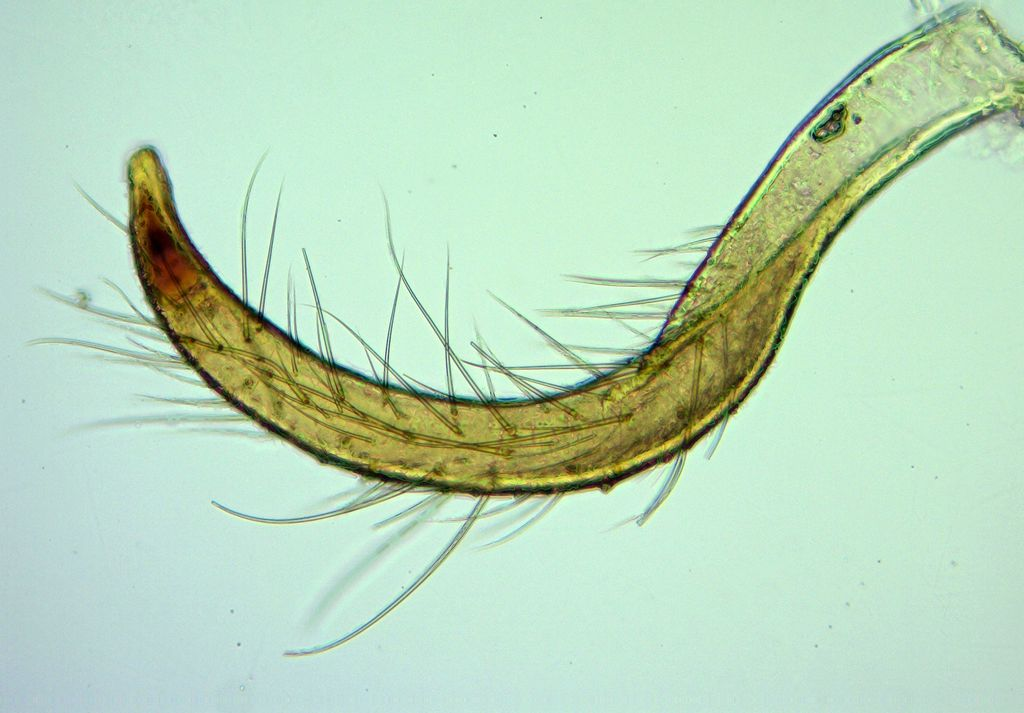
*Myiophanes greeni*, paramere in lateral view.

Mid and hind legs mostly pale brown; coxae shining white with lateral and ventral brown patches; femorotibialjunctions creamy white; femora slightly dark brown before their apical white annuli, tibiae slightlydark brown distad to basal white annuli, rest pale brown. Hind legs much longer than mid legs. Tibiaeextremely long. Tarsus very small and pale in all legs. Claws dark brown. All legs longly pilose.Aedeagus as shown in Fig. 8​. Parameres symmetrical, curved, sickle-shaped, with sparse, long setae, apex beak-like (Fig. 9).

#### Distribution

Sri Lanka (Wygodzinsky 1966; Rédei 2005) and India (present new record)

## Discussion

Rédei (2005) described two new species of the genus *Myiophanes*, one from Bangalore, India (*M. zebrina* Rédei, 2005) and the other from Pakistan (*M. incompta* Rédei, 2005), both of which are very similar, in overall coloration, to *M. greeni*, and also briefly reviewed distribution of various species of *Myiophanes*. These aspects are therefore not reiterated here. It is interesting that the pygophore, dissected phallus and paramere illustrated by Rédei (2005) for *M. zebrina* are very similar to what we have shown here, only pronotal coloration is different in all three species. Comparison with the image of the type of *M. greeni* (preserved in NHM, London) indicates that the specimen from Satara is almost the same and differs only slightly in pronotal coloration. The description given by Distant (1904) and the image of the type allow us conclude that our Satara specimen represents *M. greeni*. In personal communication, Dávid Rédei also affirmed our opinion.

Additional material of the species, from Satara and Bangalore in India, and type locality in Sri Lanka, is necessary for true comparison and resolving this issue further. Molecular work may be helpful in future to find about genetic similarity-differences among all these closely resembling species.

## Supplementary Material

XML Treatment for Myiophanes (Myiophanes) greeni

## Figures and Tables

**Figure 2a.**
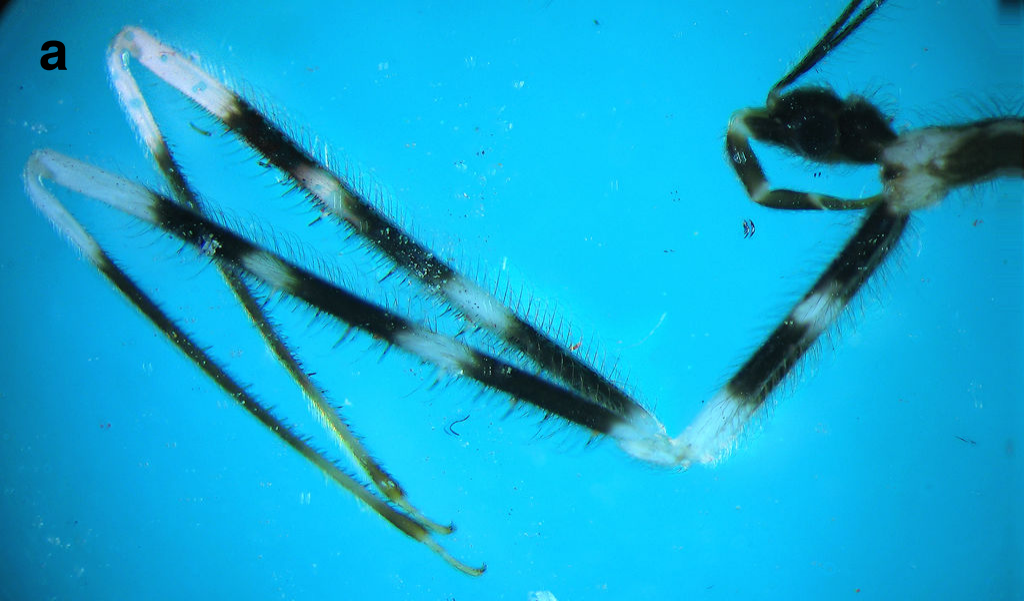
Foreleg, lateral view.

**Figure 2b.**
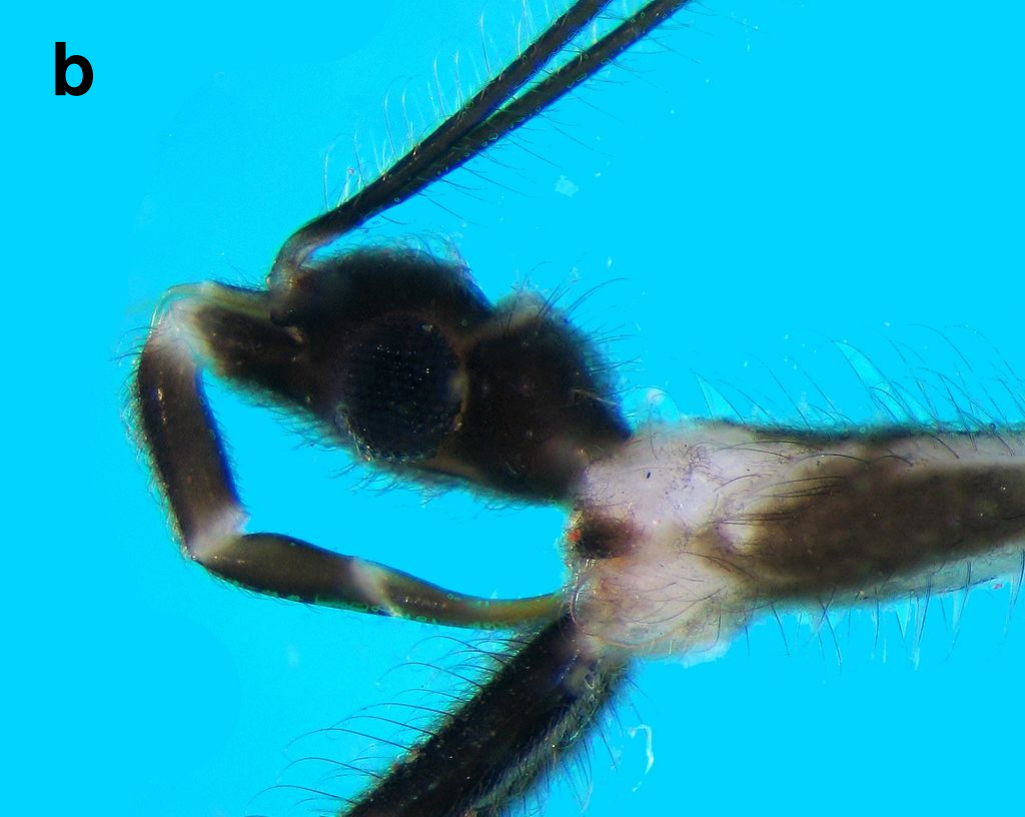
Head, lateral view.

**Figure 3a.**
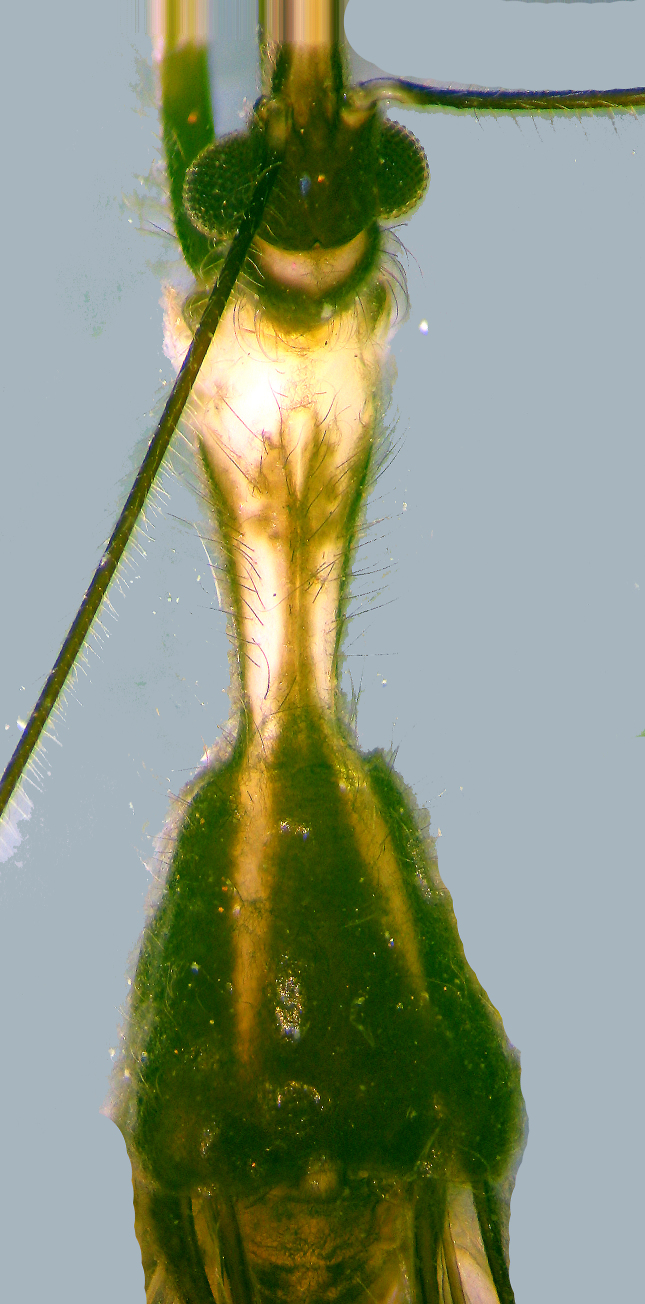
Prothorax, dorsal view.

**Figure 3b.**
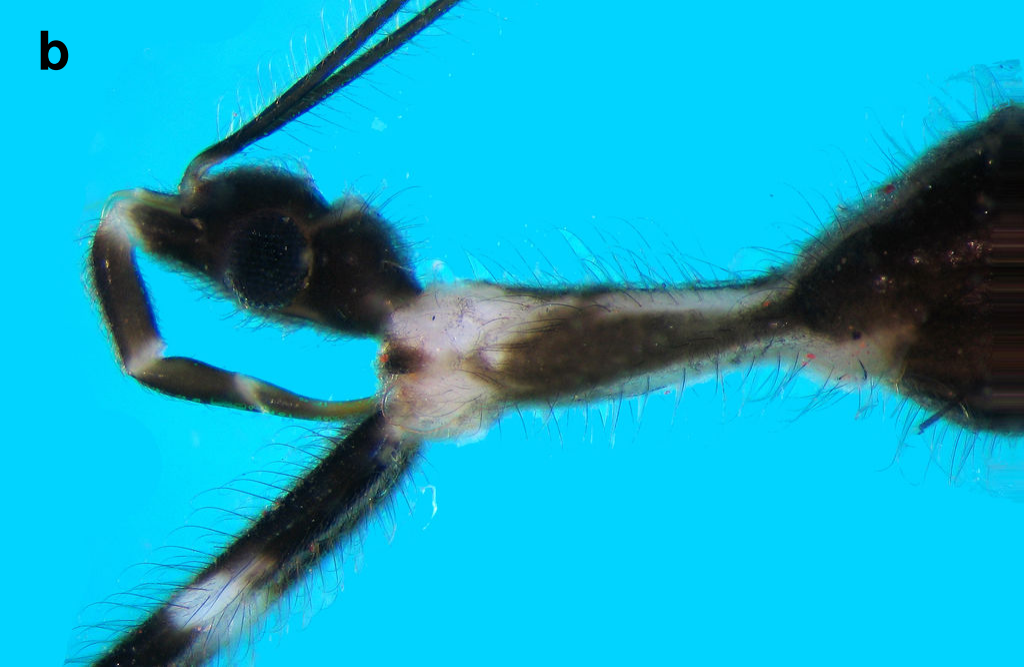
Prothorax, lateral view.

**Figure 5a.**
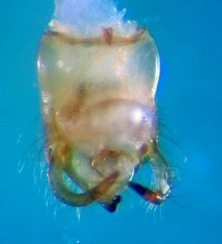
Pygophore, dorsal view.

**Figure 5b.**
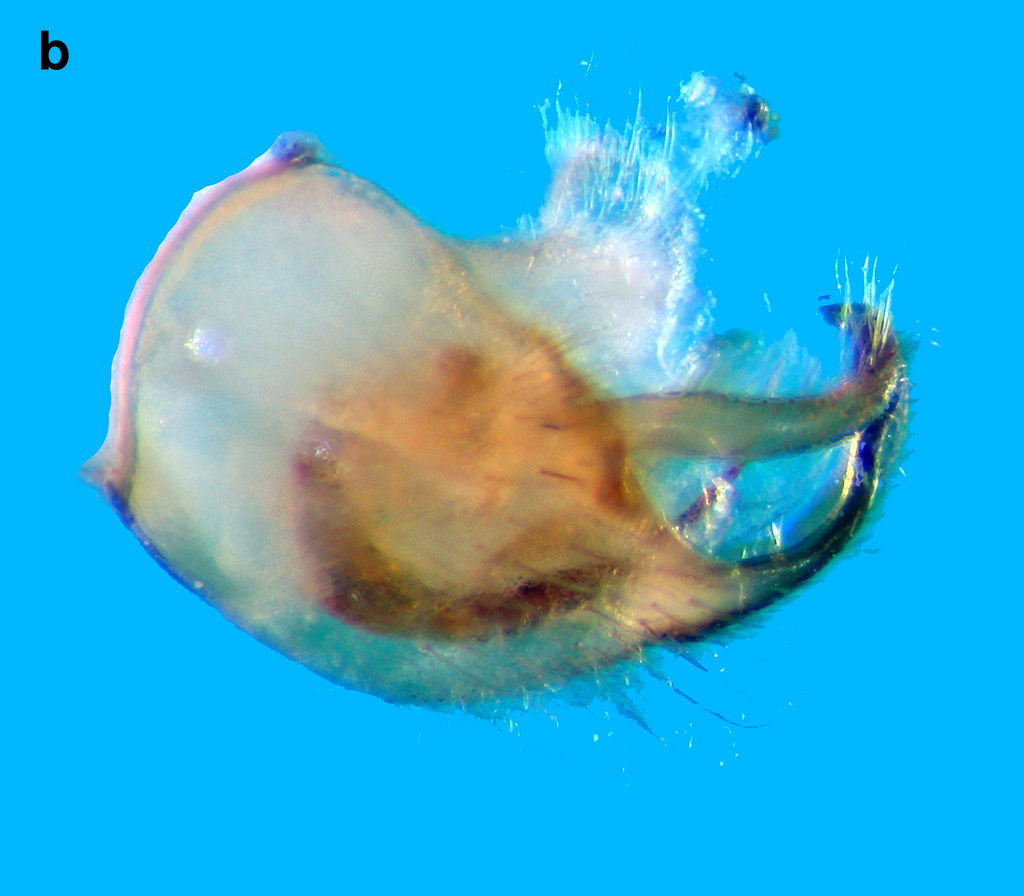
Pygophore, lateral view.

**Figure 7a.**
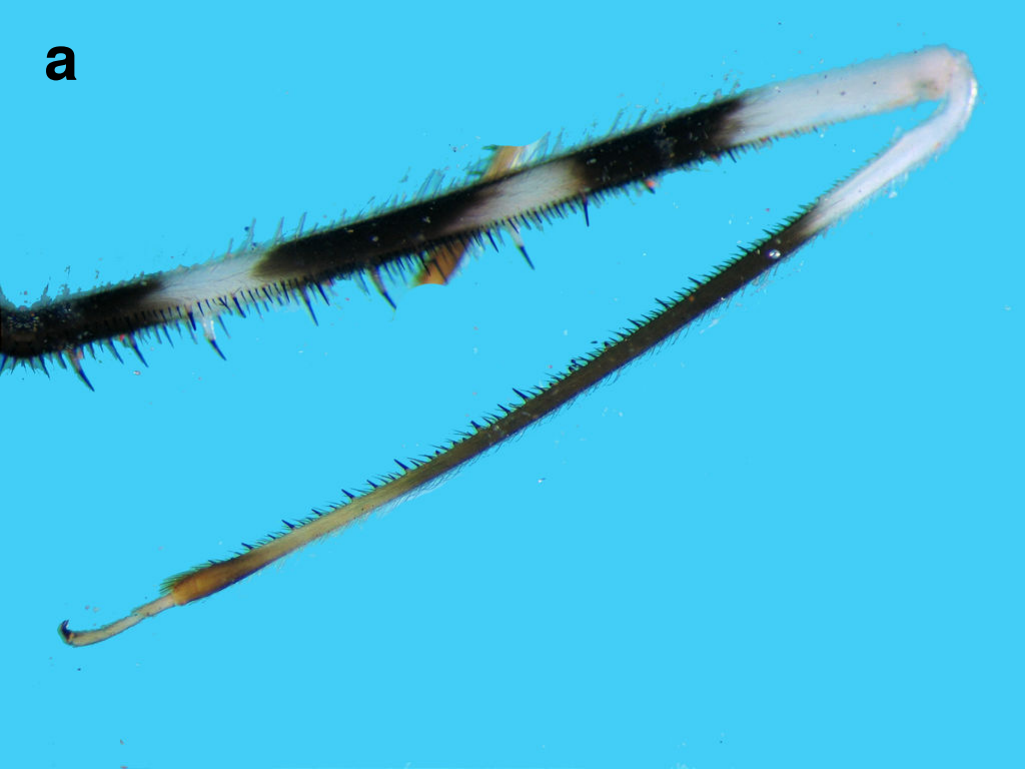
Part of fore femur, tibia and tarsus.

**Figure 7b.**
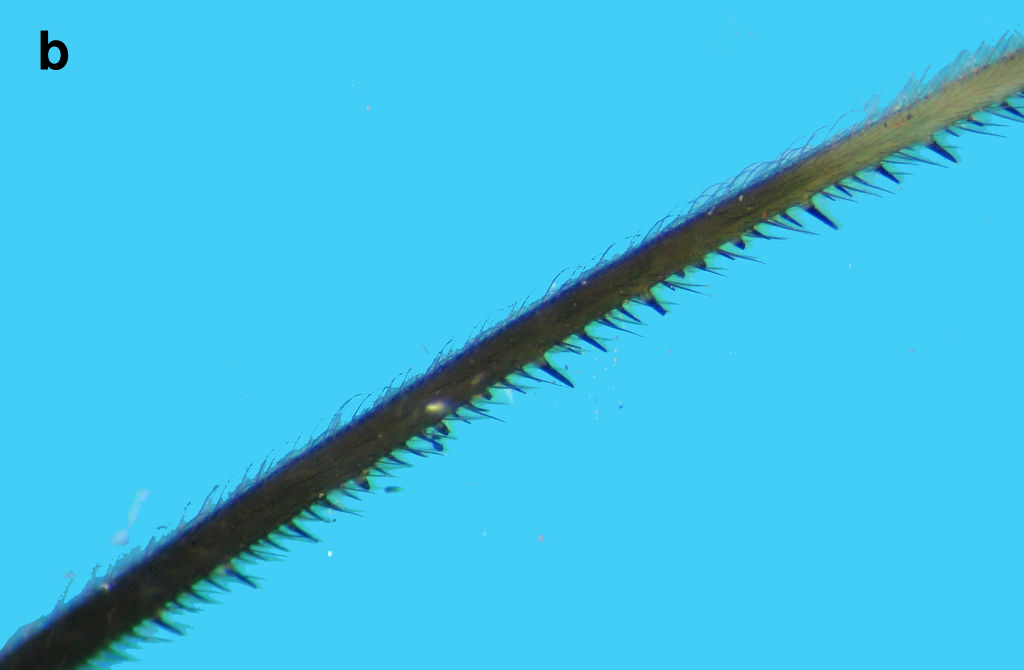
Spiniform processes of tibia.

**Figure 8a.**
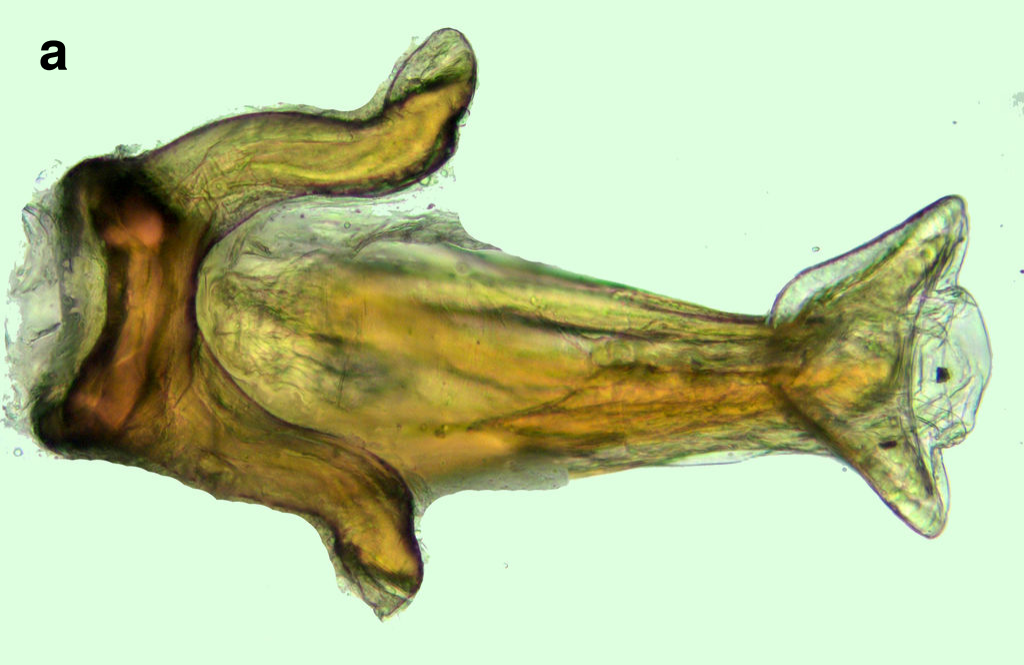
Aedeagus (endosoma in repose, not everted), dorsal view.

**Figure 8b.**
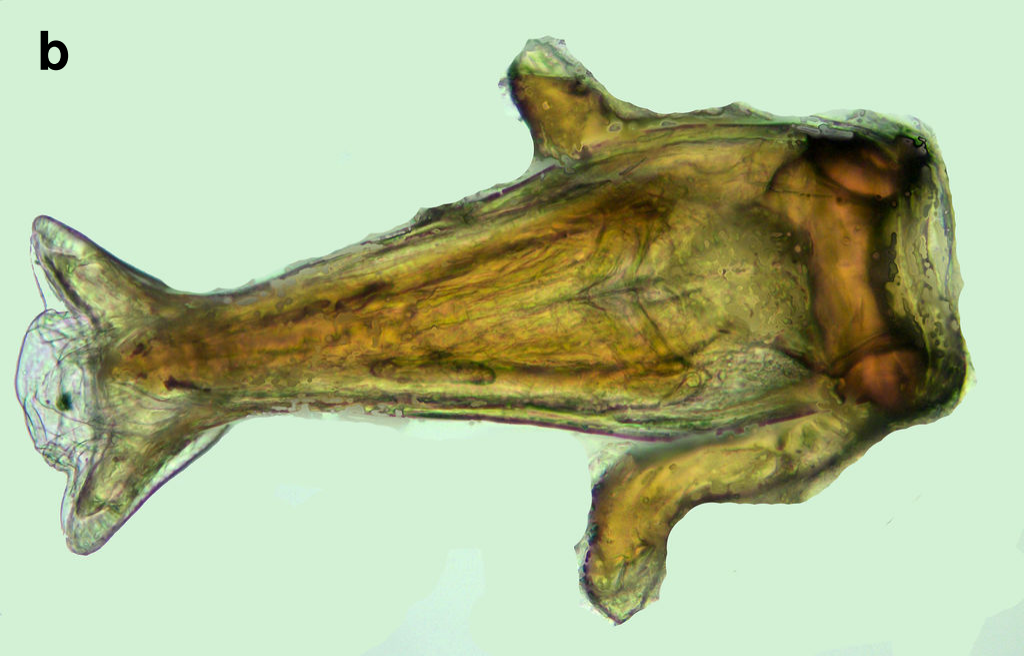
Aedeagus (endosoma in repose, not everted), ventral view.

## References

[B2736811] Ambrose D. P. (2006). A Checklist of Indian Assassin bugs (Insecta: Hemiptera: Reduviidae) with taxonomic status, distribution and diagnostic morphological characteristics. Zoos’ Print Journal.

[B2736750] Distant W. L. (1904). Rhynchota - (Heteroptera) [part]. The fauna of British India, including Ceylon and Burma. Taylor&Francis, London.

[B2736772] Rédei D. (2005). New and little-known thread-legged assassin bugs from Central and South Asia (Heteroptera: Reduviidae: Emesinae). Folia Entomologica Hungarica.

[B2736762] Wygodzinsky P. (1966). A monograph of the Emesinae (Reduviidae, Hemiptera). Bulletin of the American Museum of Natural History.

